# Socioeconomic inequality in overweight/obesity among US children: NHANES 2001 to 2018

**DOI:** 10.3389/fped.2023.1082558

**Published:** 2023-02-16

**Authors:** Stanislav Seydou Traore, Yacong Bo, Guangning Kou, Quanjun Lyu

**Affiliations:** ^1^ Department of Nutrition and Food Hygiene, College of Public Health, Zhengzhou University, Zhengzhou, China; ^2^ Jockey Club School of Public Health and Primary Care, The Chinese University of Hong Kong, Hong Kong SAR, China; ^3^ Centre of Sport Nutrition and Health, School of Physical Education, Zhengzhou University, Zhengzhou, China; ^4^ Department of Nutrition, the First Affiliated Hospital of Zhengzhou University, Zhengzhou, China

**Keywords:** overweight & obesity, children under five, concentration index, slope index of inequality, NHANES

## Abstract

**Background:**

Previous research has found that the prevalence of childhood overweight/obesity varies depending on household income, ethnicity, and sex. The goal of our research is to examine changes over time in socioeconomic inequality and the prevalence of overweight/obesity among American children under five by sex and ethnicity.

**Methods:**

This cross-sectional analysis used data from the National Health and Nutrition Examination Surveys (NHANES) collected from 2001–02 to 2017–18. Overweight/obesity in children under five [Body Mass Index (BMI)-for-age z-score >2 standard deviations] was defined according to the World Health Organization (WHO) growth reference standard. The slope inequality index (SII) and the concentration index (CIX) were used to measure the socioeconomic inequality in overweight/obesity.

**Results:**

Between 2001–02 and 2011–12, childhood overweight/obesity in the United States decreased from 7.3% to 6.3%, and had increased to 8.1% by 2017–18. However, this pattern varied widely by ethnicity and sex. For both the 2015–16 and 2017–18 surveys, overweight/obesity was more concentrated in the poorest household quintile for overall Caucasian children ((SII = −11.83, IC 95% = −23.17, −0.49 and CIX = −7.368, IC 95% = −13.92, −0.82) and (SII = −11.52, IC 95% = −22.13, −0.91 and CIX = −7.24, IC 95% = −13.27, −1.21), respectively) and for males of other ethnicities [(SII = −13.93, IC 95% = −26.95, −0.92) and CIX = −8.55, IC 95% = −0.86, −16.25] and (SII = −21.19, IC 95% = −40.65, −1.74) and CIX = −13.11, IC 95% = −1.42, −24.80), respectively). In the last three surveys, overweight/obesity was also more concentrated in the poorest household quintile for the overall children of other ethnicities. With the exception of African American females in the 2013–14 survey, for whom overweight/obesity was significantly concentrated in a quintile of the richest households (SII = 12.60, 95% CI = 0.24, 24.97 and CIX = 7.86, 95% CI = 15.59, 0.12); overweight/obesity was found to be concentrated in the richest household quintile for overall African American children, but not significantly so.

**Conclusions:**

Our findings give an update and reinforce the notion that overweight/obesity in children under the age of five has increased and that related wealth inequalities are a public health problem in the United States.

## Introduction

Overweight/Obesity among children in the United States has increased dramatically over the last three decades (1, 2), rising from 5% in 1971–1974 to 13.4% in 2017–2018 among children aged 2–5 years (3). Previous research has found that the prevalence of childhood obesity by family income varies by ethnic group, and sex (4, 5). During 2017–2018, Obesity was most prevalent in Hispanic children aged 2–19 years (25.6%), followed by African Americans (24.2%), Caucasians (16.1%), and Asians (8.7%) (6), and was greater in low-income households (18.9%) than in high-income households (10.9%) (7, 8). Studies of National Health and Nutrition Examination Surveys (NHANES) data have used the concentration index to quantify socioeconomic inequalities in overweight/obesity among adolescents (5) and adults (9). Several authors have recommended the use of inequality measures to study social inequalities in health (10–12). Unlike the standard method (regression analysis), it has the advantage of using the entire population to overcome the effect of small sample sizes in some subgroups and allows comparisons over time (13, 14). Given the complexity of obesity (15), which has many contributing elements such as biological, behavioral, genetic, environmental, and developmental components (16), it is crucial to identify the presence and evolution of socioeconomic inequalities between ethnicity groups over time. Therefore, this study aimed to assess the levels and changes over time in socioeconomic inequalities of overweight/obesity among American children under the age of five by sex and ethnicity, using NHANES data from 2001–02 to 2017–18.

## Materials and methods

### Data sources and study design

Our analyses focused on data from children under the age of five who took part in the NHANES from 2001–02 to 2017–18, a total of nine surveys. The NHANES is a study conducted by the Centers for Disease Control and Prevention (CDC) that gathers cross-sectional data on the health, nutrition, and health behavior of the civilian noninstitutionalized population in the United States. The survey used a multistage stratified cluster probability sampling approach, involving careful selection by geographic region, home composition, and person, to ensure a nationally representative sample. Participants in each survey were invited to engage in an interview in their homes, followed by physical examinations in a mobile examination center (MEC). The revision of subgroup proportions within the total population was taken into account during sample weighting methods (17). The databases and detailed information on the sampling procedure are freely available on the CDC website (18). In a brief, we only included participants who had available anthropometric measurements of height/length and body weight. To prevent the effect of unhealthy weights for length/height (19, 20), children with a BMI-for-age z-score of less than −6.0 SD or greater than +6.0 SD ratio were excluded from the analysis (21). Children with missing values of the family income/poverty were also excluded from the analysis. In compliance with the NHANES protocol, informed consent was obtained from the parents/legally authorized representatives of subjects that are under 16. The study was approved by the National Center for Health Statistics' institutional review board (17, 22).

### Malnutrition indicators

The BMI-for-age z-score for children was calculated using the WHO tools, which are freely available online (23). We categorized children as overweight/obesity or not based on whether their body mass index (BMI) for their age z-score was above two standard deviations (SD) of the WHO growth reference standard (19).

### Ethnicity

After obtaining a list with an open-ended response, data on race/ethnicity was obtained from a family member's self-report, and the variable was divided into three categories as follows: Caucasians (Non-Hispanic Whites), African American (Non-Hispanic African Americans), and other ethnicity (including Mexican Americans, other Hispanics and other races) (17). Because of the small sample sizes in the NHANES surveys, the ethnic groups “other Hispanics” and “other races (including multiracial)” were added to “Mexican Americans”.

### Indicator of socioeconomic status (wealth Index)

The poverty-income ratio (PIR) was used as the indicator of socio-economic status. The family income-to-poverty ratio was calculated by the Census Bureau by dividing total annual household income (adjusted for inflation) by the poverty line, while controlling for family size, year and state (24). The variables from the nine surveys were summed and divided into five quintiles with an equal number of participants in each quintile. The division was adjusted for ethnicity and survey years. The labels for the quintiles are as follows: Quintile 1 (Q1): poorest; Quintile 2 (Q2): poor; Quintile 3 (Q3): middle; Quintile 4 (Q4): rich; and Quintile 5 (Q5): richest.

### Other demographic variables

Participants' ages were separated into six groups: less than 6 months, 6–11 months, 12–23 months, 24–35 months, 36–47 months and 48–59 months. The participants were categorized according to their sex into two groups: Male and Female.

### Statistical analysis

The descriptive analysis was used to summarize the sample's characteristics. Graphical methods were used to visualize how overweight/obesity in children differed by ethnicity over time. The probability values were determined using column proportion tests. We used Equiplot charts to illustrate the prevalence of overweight/obesity in each wealth quintile by sex and ethnicity for each survey year. The line between the prevalence of the first and last wealth quintiles shows the degree of dispersion; the longer the line, the greater the socioeconomic dispersion. The slope inequality index (SII) and concentration index (CIX) were then calculated for each survey year and group data to estimate wealth inequality in overweight/obesity by ethnicity (25). To analyze health inequalities across wealth quintiles, it is recommended that both absolute and relative measures of inequality be used simultaneously (26). When the results of both inequality measures are significant, inequality between quintiles is asserted. The SII is a weighted, absolute measure of inequality that uses a logistic regression to represent the absolute difference in estimated values of a health indicator between the poorest and richest quintiles, while controlling for all other wealth quintiles (25, 27). The CIX is a weighted, relative measure of inequality that is related to the Gini coefficient. It calculates the magnitude and direction of health inequality. It is defined as twice the area between the concentration curve and the diagonal, ranging from 1 to 1. The concentration curve represents the cumulative percentage of the health variable relative to the cumulative percentage of the sample, ranked by socioeconomic status from the most disadvantaged group to the most advantaged group (9, 13). In the CIX analysis, we used the Erreygers correction, as suggested by other researchers who have studied health inequalities (28–30). Understanding and interpreting the SII and CIX is easy. With values ranging from −1 to 1, the SII or CIX is a composite description of inequality across the population. The index is zero when there is perfect equality. When the values are negative, children from the poorest quintile are most affected by overweight/obesity; When the values are positive, children from the richest quintile are most affected by overweight/obesity. The magnitude of the index reveals the level of inequality (25). The CIX allowed comparisons of wealth inequality between surveys for annualized change, plots of the CIX indices and 95% confidence intervals were made. When the CIX value of the annualized change is positive or negative, wealth inequality is said to have decreased or increased, respectively. The analyses were weighted and found to be appropriate for the complex NHANES survey design. Probability values for statistical tests, where 2-sided *p*-values <0.05 were considered significant. Analyses were performed using Stata (STATA Corp., LP, College Station, Texas), and graphical representations were created using GraphPad Prism 9.

## Results

Analyses of pooled data from NHANES 2001–02 through 2017–18 (Table 1) revealed that children aged 2 years and older made up the majority of respondents in all ethnic groups; Males outnumbered females (50.9% and 49.1%, respectively). Pooled data covering 2001–2018 showed that Caucasians had a prevalence of 6.7%, African Americans had a prevalence of 8.6%, and other ethnicities had a prevalence of 8.0%.

**Table 1:** Sociodemographic characteristics of children under five years of age by ethnicity, NHANES 2001–2018.

	Caucasian		African American		Others		Overall	
	*N*	%	*N*	%	*N*	%	*N*	%
Participants	3,635	100	2,544	100	4,954	100	11,133	100
**Age in month**								
<6	537	8.6^B^	278	8.5	712	8.7^AB^	1,527	8.6
6–11	610	10.1^BC^	311	9.2	833	10.1^B^	1,754	10.0
12–23	739	20.1^B^	538	19.2	1,043	20.8^AB^	2,320	20.2
24–35	755	20.8^BC^	542	19.5^C^	914	18.6	2,211	20.0
36–47	481	19.4	429	21.5^AC^	693	19.7^A^	1,603	19.8
≥48	513	21.0	446	22.2^A^	759	22.1^A^	1,718	21.5
**Sex**								
Male	1,898	51.0^C^	1,306	51.1^AC^	2,470	50.6	5,674	50.9
Female	1,737	49.0^B^	1,238	48.9	2,484	49.4^AB^	5,459	49.1
**Wealth Quintiles**								
Poorest	1,113	19.7	515	20.1^AC^	1,094	20.0^A^	2,722	19.8
Poor	782	20.2^BC^	520	20.0^A^	1,080	19.8	2,382	20.1
Middle	622	20.2^BC^	496	19.6	993	19.9^B^	2,111	20.0
Rich	557	19.2	517	20.0^C^	924	20.1^AB^	1,998	19.6
Richest	561	20.6^BC^	496	20.3	863	20.2	1,920	20.5
Overweight (%)	252	6.7	218	8.6^A^	488	10.0^AB^	958	8.0
**Surveys**								
2001–02	427	9.9^C^	333	9.9^C^	479	8.0	1,239	9.3
2003–04	411	11.6^C^	373	11.8^AC^	557	9.9	1,341	11.1
2005–06	445	12.5^BC^	346	11.9^C^	703	10.4	1,494	11.8
2007–08	457	11.6^C^	251	12.8^AC^	591	11.4	1,299	11.7
2009–10	474	12.1^B^	223	11.7	616	12.6^AB^	1,313	12.2
2011–12	263	10.7	339	12.5^AC^	574	12.1^A^	1,176	11.4
2013–14	378	10.9^B^	257	10.7	543	12.4^AB^	1,178	11.3
2015–16	384	10.2^B^	238	10.0	537	12.2^AB^	1,159	10.8
2017–18	396	10.5^B^	184	8.7	354	10.9^AB^	934	10.4

N, number of participants; %, weighted percentage; Others, other ethnicities; Overall, all ethnic groups gathered. Results are based on two-tailed tests. For each significant pair, the key from the category with the smallest proportion of columns appears in the category with the largest proportion of columns. Level of significance for uppercase letters (A, B, C): *p*-value <0.05 from columns proportion test.

**Figure 1:**
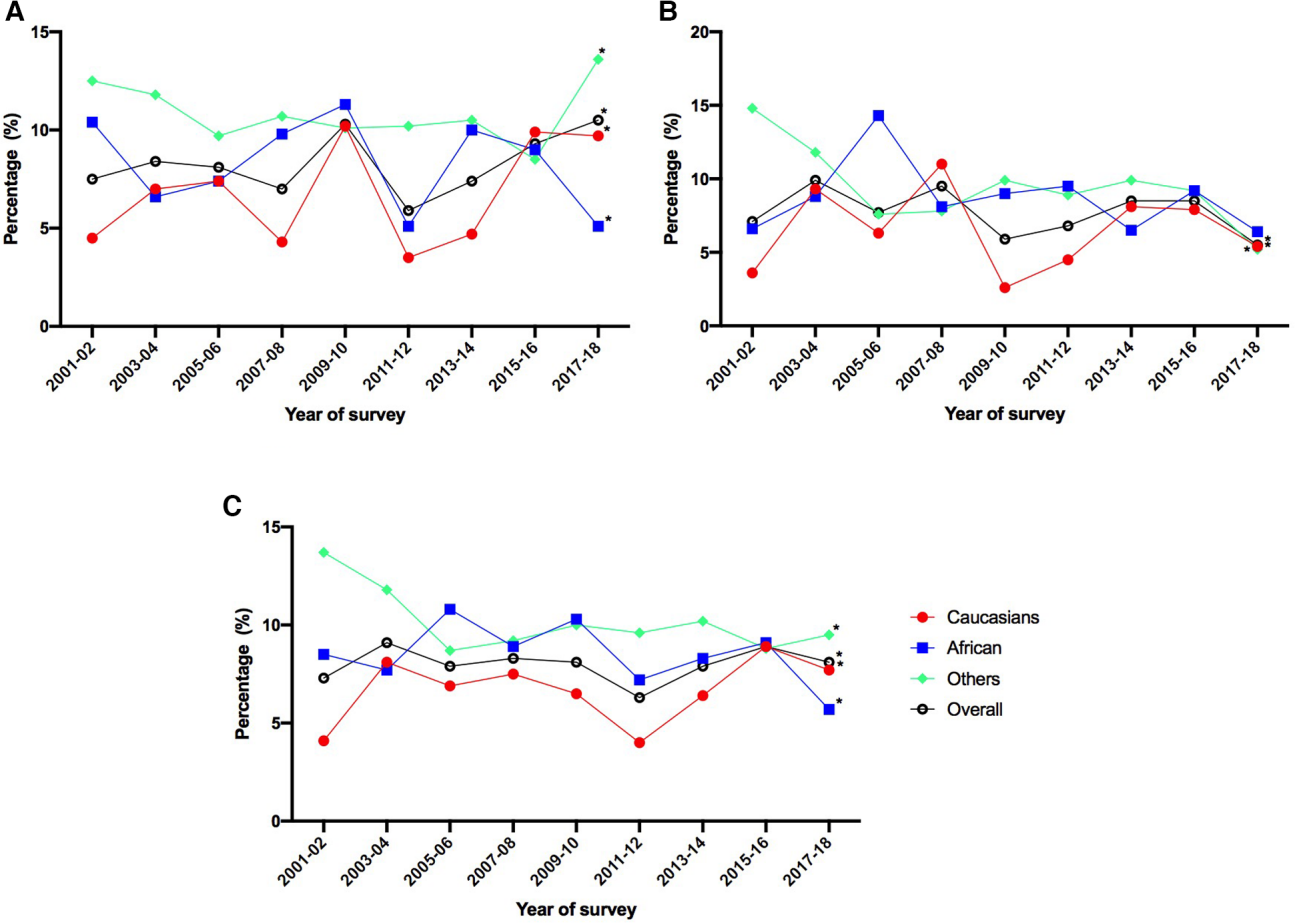
Overweight/obesity prevalence in children by ethnic group and survey year for each sex. Caucasian: Caucasian, African: African American, Others: other ethnicities, Overall: All participants gathered. **A: Male, B: Female, C: Overall**. *p-value <0.05 from column proportion test between 2001–02 and 2017–18.

Between 2001–02 and 2011–12, the prevalence of childhood overweight/obesity in the United States decreased from 7.3% to 6.3%, and had increased to 8.1% by 2017–18. However, this pattern varied widely by ethnicity and sex (Figure 1). From 2011–12 to 2017–18, the prevalence of overweight/obesity in males has steadily increased from 5.9% to 10.5%. While the prevalence of overweight/obesity among females began to rise in 2009–2010 (5.9%), stabilized between 2013–14 and 2015–16 (8.5%), and then decreased to 5.5% in 2017–18.

**Figure 2:**
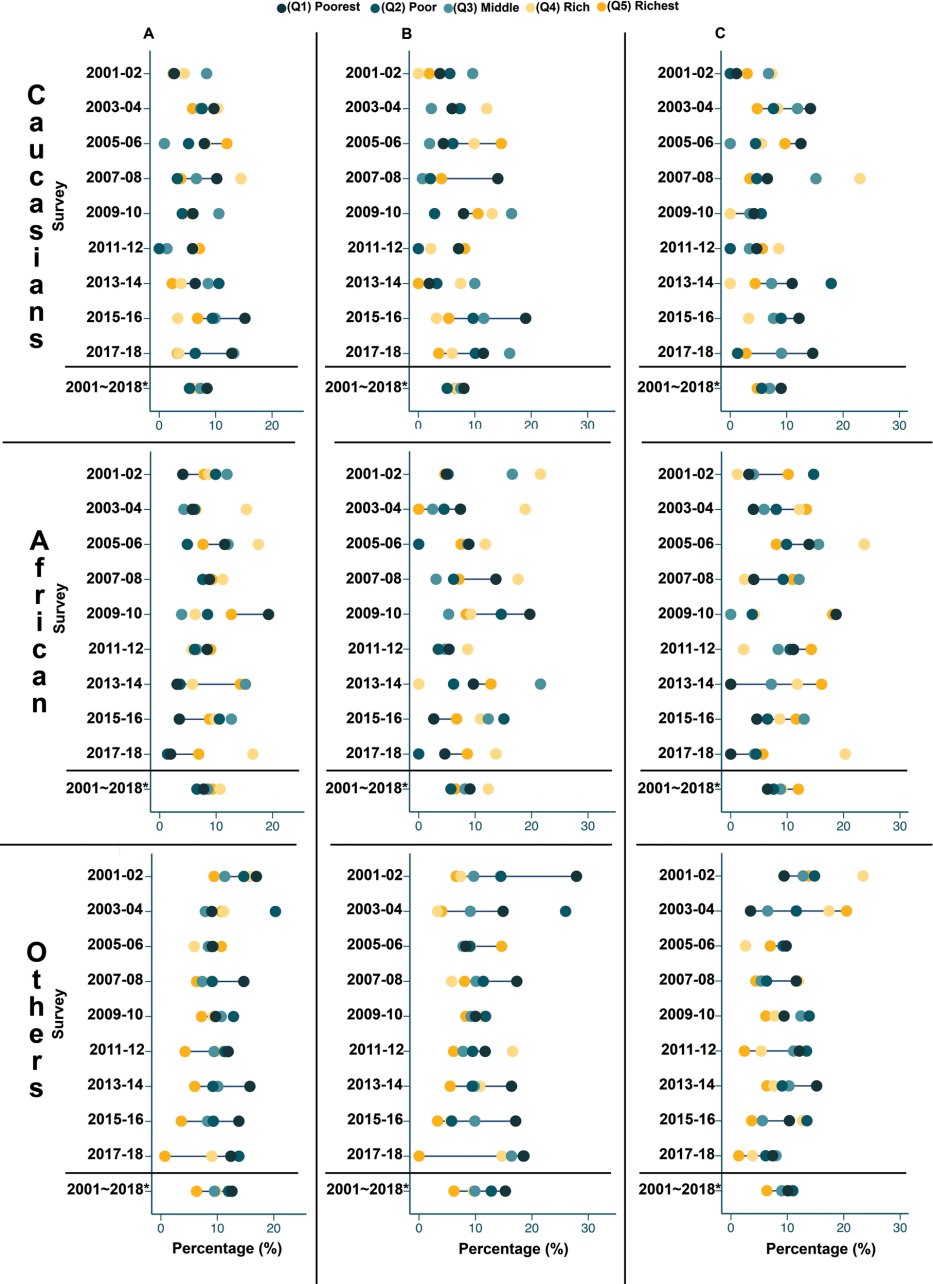
Prevalence of overweight/obesity by wealth quintile, for each survey year and for pooled data. Caucasian: Caucasian, African: African American, Others: other ethnicities, Overall: All children from the same ethnic group gathered. (Q1): Quintile 1, (Q2): Quintile 2, (Q3): Quintile 3, (Q4): Quintile 4, (Q5): Quintile 5. **A: Overall, B: Male, C: Female**.

The greatest dispersions between the richest and poorest quintiles were observed among males of other ethnicities (Figure 2), with the poorest quintile becoming more overweight/obesity in recent years (from 10.0% to 8.5% between 2009–10 and 2017–18) and the richest quintile becoming less overweight/obesity (from 8.3% to 0.3% between 2009–10 and 2017–18). Between 2015–16 and 2017–18, the gap between the richest and poorest Caucasian females widened, with an increase in overweight/obesity among the poorest and a decrease among the richest (from 12.2% to 14.6% and 8.0% to 2.8%, respectively).

**Figure 3:**
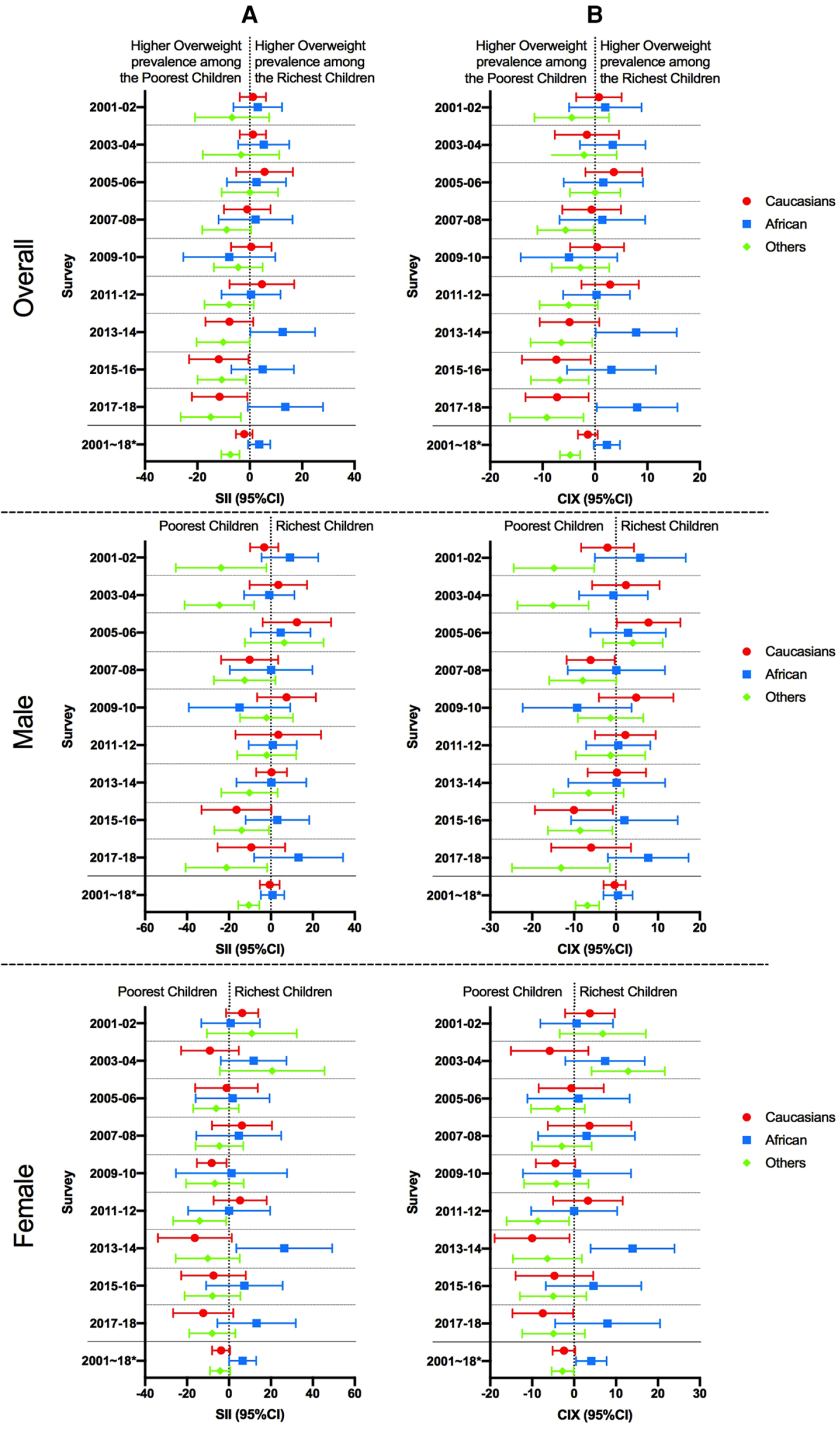
Absolute and relative wealth inequality of overweight/obesity prevalence among children by sex for each year of survey and pooled data. CIX: concentration Index, SII: Slope Index of Inequality. Caucasian: Caucasian, African: African American, Others: other ethnicities. CI: confidence interval.*: pooled data. (**A**) Relative inequality, (**B**) Absolute inequality.

Figure 3 plots the SII and CIX results, along with their 95% confidence intervals. For both the 2015–16 and 2017–18 surveys, the SII and CIX were significantly lower than 0 among the overall Caucasian children ((SII = −11.83, IC 95% = −23.17, −0.49 and CIX = −7.368, IC 95% = −13.92, −0.82) and (SII = −11.52, IC 95% = −22.13, −0.91 and CIX = −7.24, IC 95% = −13.27, −1.21), respectively) and among males of other ethnicities [(SII = −13.93, IC 95% = −26.95, −0.92) and CIX = −8.55, IC 95% = −0.86, −16.25] and (SII = −21.19, IC 95% = −40.65, −1.74) and CIX = −13.11, IC 95% = −1.42, −24.80), respectively). Indicating a higher concentration of overweight/obesity in a quintile of the poorest households. With the exception of African American females in the 2013–14 survey, for whom SII and CIX were significant (SII = 12.60, 95% CI = 0.24, 24.97 and CIX = 7.86, 95% CI = 15.59, 0.12), SII and CIX were non-significantly larger than 0 in the overall African American children, indicating a higher concentration of overweight/obesity in a quintile of the richest households.

**Figure 4:**
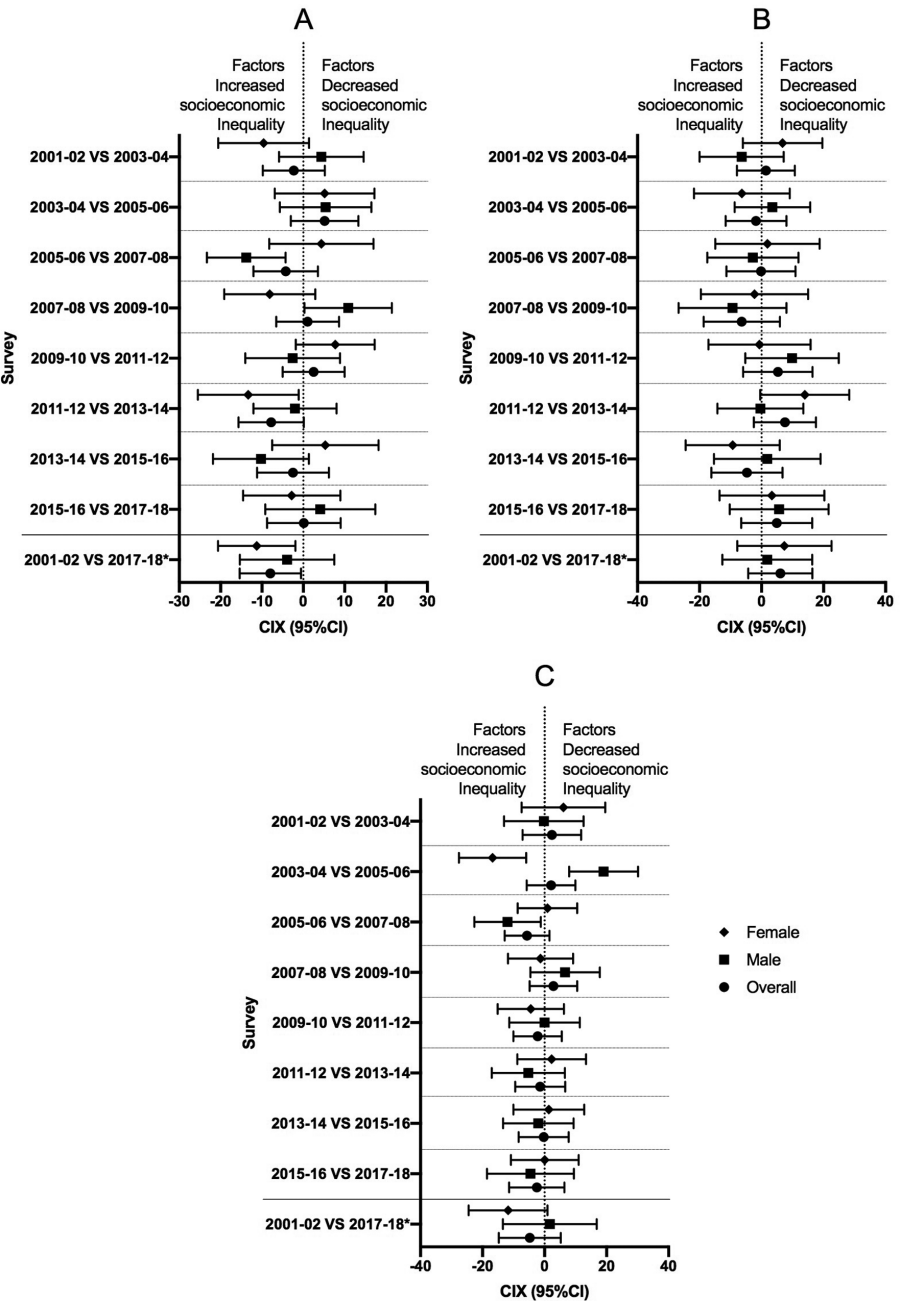
Annualized changes of relative wealth inequality in the overweight/obesity prevalence among children by sex. CIX: concentration Index. **A: Caucasian, B: African American, C: Other ethnicities**, CI: confidence interval. *: comparison of inequality between 2001–02 and 2017–18.

Annualized changes in relative wealth inequality are plotted in Figure 4. The annualized relative changes in the CIX among African Americans for both males and females are not significantly different from 0 in terms of wealth disparity. indicating a negligible difference between years. The CIX of wealth inequality for overweight/obesity showed annualized relative decreases between 2005–06 and 2007–08 among Caucasian males (CIX = −13.81, 95% CI = −4.30, −23.32), and between 2011–12 and 2013–14 among Caucasian females (CIX = −13.31, 95% CI = −1.11, −25.52), indicating an increase in wealth inequality. Between 2003–04 and 2005–2006, the CIX for overweight showed an annualized relative decrease among females of other ethnicities (CIX = −16.77, 95% CI = −5.94, −27.61), indicating an increase in wealth inequality. In contrast, a decrease in the CIX for males over the same period (CIX = −11.93, 95% CI = −1.20, −22.65) was followed by an increase between 2005–06 and 2007–2008 (CIX = 19.0, 95% CI = 30.14, 7.94), indicating a significant increase followed by a significant decrease in wealth inequality.

## Discussion

The concentration index (CIX) technique has been applied in the area of adolescent overweight in the 1999–2002 NHANES surveys (5). The present study focused on children under 5 years of age and used the CIX technique to examine trends in socioeconomic inequality of overweight/obesity from the 2001–02 to 2017–18 surveys. The findings of this study showed that the prevalence of childhood overweight/obesity in the United States declined between 2001–02 and 2011–12, then increased up until 2017–18. This pattern varied greatly by ethnic group and by sex. Wealth inequalities in overweight/obesity have emerged significantly in recent surveys, with the poorest children of Caucasians and other ethnicities bearing the brunt of the burden.

Our findings are consistent with previous research that found a significant decrease in the prevalence of overweight/obesity among children aged 2–5 years between 2003–04 and 2011–12 (1, 31) and reported that overweight/obesity varies by ethnic groups, with African Americans, American Indians, and Mexican Americans being more affected than non-Hispanic Whites (4, 32). There are hereditary risks for childhood obesity that have been described. When one or both parents are overweight/obese, the risk of having an overweight/obese child increases by 2–3 times or 15 times, respectively (33). The authors identified a number of factors that have contributed to the obesity epidemic among African Americans, including incorrect BMI classification, which may have led to 30%–60% of African American children being classified as overweight/obese (34). In addition, racial segregation (35, 36) and racism (37, 38) may prevent African Americans from escaping obesogenic factors associated with their environments (39).

Our findings are consistent with previous research suggesting that obesity is not always concentrated in low-income households (2, 5, 7, 40). Children from low-income Caucasian and Hispanic households were most likely to be overweight or obese. However, this relationship did not apply to African American children (41, 42). Although more African American children in the richest households were overweight or obese than in the poorest, the difference was only marginally significant (with the exception of 2013–14).

Our findings showed that among Caucasians and other ethnicities, wealth inequalities in childhood overweight/obesity emerged significantly in 2015–16 and 2017–18, which is consistent with previous research indicating that the strength of the relationship between income and the prevalence of overweight/obesity has increased over time (2); whereas other studies have found this relationship to be absent (5) or inconsistent across racial/ethnic groups (40).

The current study also found that the prevalence of overweight/obesity among male children of other ethnicities revealed persistent wealth inequalities between 2001–02 and 2017–18. These inequalities were due in part to a continual rise in the prevalence of overweight in the poorest group since 2009–10, while a continual decline had been seen in the richest group over the same time period. A stronger association between family income and childhood overweight was observed among Mexican-American children aged 2–5 years; however, the correlation did not differ significantly between males and females, independent of racial/ethnic groups (40). Another study found an inverse association among Mexican-American females only (43).

In the 2001–02 and 2003–04 surveys, overweight/obesity was concentrated among females of other ethnicities in the richest group, but the direction completely changed in 2005–06, with the poorest being the most affected. These findings are partially consistent with a previous study, which found that the direction of the relationship between income and overweight/obesity shifted for all children after 2004 (40).

Our findings are consistent with previous findings that overweight/obesity was more concentrated among African American males and females in the richest group. In contrast, our findings from 2001 to 2018 pooled data revealed that this relationship was significant only in African American females. It has been reported that obesity is increasingly linked to poverty, food insecurity (44) and the risk of being passed on to future generations. Authors have reported that childhood obesity is strongly related to the socioeconomic status of their parents, and that countries with the greatest wealth inequality have higher rates of childhood obesity (45). Mothers are less likely to breastfeed when they are obese and of low socioeconomic status. Thus, non-breastfed infants are more likely to have unhealthy eating habits, become obese and have delayed cognitive development by age 3 (45). On the other hand, evidence linking food insecurity and obesity is limited to adult women in developing countries (46).

The environment in which children and adolescents from low-income households grow may also contribute to their increased risk of being overweight or obese (7, 47, 48). Children from low-income or racial/ethnic minority families most frequently reside in underdeveloped areas with few stores (49), which restricts their access to nutritious foods like fruits and vegetables (50, 51) and encourages the consumption of inexpensive, high-calorie, high-fat foods (52). Moreover, these areas frequently lack parks for outdoor recreation (53). Debates are still ongoing about the relative culpability of energy intake vs. energy expenditure (physical activity) in weight gain. But most studies point to overconsumption of energy-dense foods as the primary culprit, and a decrease in physical activity is unlikely to be responsible for a dramatic increase in obesity in lower socioeconomic groups (54, 55).

Given the complexities of the factors influencing childhood obesity development (53, 56–58). Multidisciplinary interventions addressing individual, family, economic, environmental, social, and cultural barriers are required to prevent the progression of obesity (59).

To our knowledge, we are among the first to investigate wealth inequalities in overweight/obesity in United States children under the age of five using inequality measures proposed by the authors (5, 60). The advantage of using the SII and CIX is particularly relevant in the current study because it takes into account the entire population to overcome the effect of small sample sizes in some subgroups (5, 61). In addition, analysis on pooled data from all 9 surveys was conducted to increase the power of the analysis.

Limitations of this study include the cross-sectional nature of the NHANES data, which does not give insight on causality or causes; income may not correlate with individuals' actual socioeconomic status (62). This would necessitate the use of a longitudinal design to establish temporal precedence, as well as the inclusion of additional environmental, behavioral, and biological measurements (63). The CDC advises utilizing WHO growth standards for children under the age of two, and CDC growth benchmarks for children two and older (64). Nonetheless, we followed the WHO growth guidelines (65). Although these standards are probably not optimum for all parts of the pediatric population, they were applied to all children worldwide, regardless of ethnicity, socioeconomic status, or food type.

## Conclusion

This study adds to previous research using the CIX technique in the domain of wealth inequality in the overweight/obese population in the United States. Overall, our findings give an update and reinforce the notion that overweight/obesity in children under the age of five has increased and that related wealth inequalities are a public health problem in the United States. In the United States, rising childhood overweight/obesity is associated with rising wealth inequality. We propose that policies and programs aimed at preventing childhood overweight/obesity should include minorities and low socioeconomic groups, but that population-based treatments should target al.l ethnic groups.

## Data Availability

Publicly available datasets were analyzed in this study. This data can be found here: https://www.cdc.gov/nchs/nhanes.

## References

[1] Ogden CL Carroll MD Lawman HG Fryar CD Kruszon-Moran D Kit BK Trends in obesity prevalence among children and adolescents in the United States, 1988–1994 through 2013–2014. JAMA. (2016) 315(21):2292–9. 10.1001/jama.2016.6361 27272581 PMC6361521

[2] Weaver RG Brazendale K Hunt E Sarzynski MA Beets MW White K. Disparities in childhood overweight and obesity by income in the United States: an epidemiological examination using three nationally representative datasets. Int J Obes. (2019) 43(6):1210–22. 10.1038/s41366-019-0331-2 PMC11460982 30718822

[3] Fryar CD Carroll MD. M.S JA. Prevalence of overweight, obesity, and severe obesity among children and adolescents aged 2–19 years: United States, 1963–1965 through 2017–2018.: Centers for Disease Control and Prevention National Center for Health Statistics (2021) [updated Updated January 29, 2021]. Available at: https://www.cdc.gov/nchs/data/hestat/obesity-child-17-18/obesity-child.htm.

[4] Anderson SE Whitaker RC. Prevalence of obesity among US preschool children in different racial and ethnic groups. Arch Pediatr Adolesc Med. (2009) 163(4):344–8. 10.1001/archpediatrics.2009.18 19349563

[5] Zhang Q Wang Y. Using concentration index to study changes in socio-economic inequality of overweight among US adolescents between 1971 and 2002. Int J Epidemiol. (2007) 36(4):916–25. 10.1093/ije/dym064 17470489

[6] (CDC) CoDCaP. Childhood Obesity Facts. Prevalence of Childhood Obesity in the United States [updated April 5, 2021]. Available at: https://www.cdc.gov/obesity/data/childhood.html.

[7] Ogden CL Carroll MD Fakhouri TH Hales CM Fryar CD Li X Prevalence of obesity among youths by household income and education level of head of household - United States 2011-2014. MMWR Morb Mortal Wkly Rep. (2018) 67(6):186–9. 10.15585/mmwr.mm6706a3 29447142 PMC5815488

[8] Tester JM Rosas LG Leung CW. Food insecurity and pediatric obesity: a double whammy in the era of COVID-19. Curr Obes Rep. (2020) 9(4):442–50. 10.1007/s13679-020-00413-x 33064269 PMC7562757

[9] Zhang Q Wang YF. Socioeconomic inequality of obesity in the United States: do gender, age, and ethnicity matter? Soc Sci Med. (2004) 58(6):1171–80. 10.1016/S0277-9536(03)00288-0 14723911

[10] Barros AJ Ronsmans C Axelson H Loaiza E Bertoldi AD França GV Equity in maternal, newborn, and child health interventions in countdown to 2015: a retrospective review of survey data from 54 countries. Lancet. (2012):379(9822):1225–33. 22464386 10.1016/S0140-6736(12)60113-5

[11] Kakwani N Wagstaff A Van Doorslaer E. Socioeconomic inequalities in health: measurement, computation, and statistical inference. J Econom. (1997) 77:87–103. 10.1016/S0304-4076(96)01807-6

[12] Wagstaff A Paci P van Doorslaer E. On the measurement of inequalities in health. Soc Sci Med. (1991) 33(5):545–57. 10.1016/0277-9536(91)90212-U 1962226

[13] O’Donnell O O’Neill S Van Ourti T Walsh B. Conindex: estimation of concentration indices. Stata J. (2016) 16(1):112–38. 10.1177/1536867X1601600112 27053927 PMC4819995

[14] Wagstaf A. The bounds of the concentration index when the variable of interest is binary,with an application to immunization inequality. Health Econ. (2005) 14(4):429–32. 10.1002/hec.953 15495147

[15] Kansra AR Lakkunarajah S Jay MS. Childhood and adolescent obesity: a review. Front Pediatr. (2021) 8:581461. 10.3389/fped.2020.581461 33511092 PMC7835259

[16] Qasim A Turcotte M de Souza RJ Samaan MC Champredon D Dushoff J On the origin of obesity: identifying the biological, environmental and cultural drivers of genetic risk among human populations. Obes Rev. (2018) 19(2):121–49. 10.1111/obr.12625 29144594

[17] Johnson CL Dohrmann SM Burt VL Mohadjer LK. National health and nutrition examination survey: sample design, 2011-2014. Vital Health Stat. (2014) 2(162):1–33. PMID: 25569458

[18] (CDC) CfDCaP. National Health and Nutrition Examination Survey. U.S.: Department of Health & Human Services.; [updated August 31, 2022 ]. Available at: https://www.cdc.gov/nchs/nhanes/.

[19] de Onis M Martorell R Garza C Lartey A, the WHO Multicentre Growth Reference Study Group. WHO child growth standards based on length/height, weight and age. Acta Paediatr. (2006) 95:76–85. 10.1080/08035320500495548 16817681

[20] Saari A Sankilampi U Hannila ML Kiviniemi V Kesseli K Dunkel L. New finnish growth references for children and adolescents aged 0–20 years: length/height-for-age, weight-for-length/height, and body mass index-for-age. Ann Med. (2011) 43(3):235–48. 10.3109/07853890.2010.515603 20854213

[21] Naga Rajeev L Saini M Kumar A Sachdev HS. Dissimilar associations between stunting and low ponderosity defined through weight for height (wasting) or body mass index for age (thinness) in under-five children. Indian Pediatr. (2022) 59(10):757–62. 10.1007/s13312-022-2617-z 35822490

[22] Zipf G Chiappa M Porter KS Ostchega Y Lewis BG Dostal J. National health and nutrition examination survey: plan and operations, 1999–2010. Vital Health Stat. (2013) 1(56):1–37. 25078429

[23] (WHO) WHO. Child growth standards. Software [updated 2022]. Available at: https://www.who.int/tools/child-growth-standards/software.

[24] How the Census Bureau Measures Poverty. Guidance for Poverty Data Users.: United States of Census Bureau; [updated November 22, 2021 ]. Available at: https://www.census.gov/topics/income-poverty/poverty/guidance/poverty-measures.html.

[25] Hosseinpoor AR Schlotheuber A Nambiar D Ross Z. Health equity assessment toolkit plus (HEAT plus): software for exploring and comparing health inequalities using uploaded datasets. Glob Health Action. (2018) 11(sup1):1440783. 10.1080/16549716.2018.1440783 29974823 PMC6041818

[26] Gebreselassie T Wang W Sougane A Boly S. Inequalities in the coverage of reproductive, maternal, newborn, and child health interventions in mali: Further analysis of the mali demographic and health surveys 2006–2018. DHS Further Analysis Report No. 135. Rockville, Maryland, USA: ICF (2020).

[27] Renard F Devleesschauwer B Speybroeck N Deboosere P. Monitoring health inequalities when the socio-economic composition changes: are the slope and relative indices of inequality appropriate? Results of a simulation study. Bmc Public Health. (2019) 19(1):662. 10.1186/s12889-019-6980-1 31146708 PMC6543610

[28] Assaf S Thomas P. Levels and trends in maternal and child health disparities by wealth and region in eleven countries with DHS surveys. DHS Comparative Reports No. 42. Rockville, Maryland, USA: ICF International (2016).

[29] Binnendijk E Koren R Dror DM. Can the rural poor in India afford to treat non-communicable diseases. Trop Med Int Health. (2012) 17(11):1376–85. 10.1111/j.1365-3156.2012.03070.x 22947207

[30] Van Malderen C Ogali I Khasakhala A Muchiri SN Sparks C Van Oyen H Decomposing Kenyan socio-economic inequalities in skilled birth attendance and measles immunization. Int J Equity Health. (2013) 12:3. 10.1186/1475-9276-12-3 23294938 PMC3547715

[31] Ogden CL Carroll MD Kit BK Flegal KM. Prevalence of childhood and adult obesity in the United States, 2011–2012. JAMA. (2014) 311(8):806–14. 10.1001/jama.2014.732 24570244 PMC4770258

[32] Goodman E Maxwell S Malspeis S Adler N. Developmental trajectories of subjective social status. Pediatrics. (2015) 136(3):E633–E40. 10.1542/peds.2015-1300 26324868 PMC4552092

[33] Whitaker RC Wright JA Pepe MS Seidel KD Dietz WH. Predicting obesity in young adulthood from childhood and parental obesity. New Engl J Med. (1997) 337(13):869–73. 10.1056/NEJM199709253371301 9302300

[34] Flegal KM Ogden CL Yanovski JA Freedman DS Shepherd JA Graubard BI High adiposity and high body mass index–forage in US children and adolescents overall and by race-ethnic group. Am J Clin Nutr. (2010) 91(4):1020–6. 10.3945/ajcn.2009.28589 20164313 PMC2844683

[35] Bower KM Thorpe RJ Jr Yenokyan G McGinty EE Dubay L Gaskin DJ. Racial residential segregation and disparities in obesity among women. J Urban Health. (2015) 92(5):843–52. 10.1007/s11524-015-9974-z 26268731 PMC4608933

[36] LaVeist T Pollack K Thorpe R Jr Fesahazion R Gaskin D. Place, not race: disparities dissipate in Southwest Baltimore when blacks and whites live under similar conditions. Health Affair. (2011) 30(10):1880–7. 10.1377/hlthaff.2011.0640 PMC6535343 21976330

[37] Williams DR. Race, socioeconomic status, and health. The added effects of racism and discrimination. Ann N Y Acad Sci. (1999) 896:173–88. 10.1111/j.1749-6632.1999.tb08114.x 10681897

[38] Hajat A Kaufman JS Rose KM Siddiqi A Thomas JC. Long-term effects of wealth on mortality and self-rated health status. Am J Epidemiol. (2011) 173(2):192–200. 10.1093/aje/kwq348 21059808 PMC3139960

[39] Swinburn B Egger G Raza F. Dissecting obesogenic environments: the development and application of a framework for identifying and prioritizing environmental interventions for obesity. Prev Med. (1999) 29(6):563–70. 10.1006/pmed.1999.0585 10600438

[40] Freedman DS Ogden CL Flegal KM Khan LK Serdula MK Dietz WH. Childhood overweight and family income. Med Gen Med. (2007) 9(2):26. PMC1994830 17955082

[41] Eagle TF Sheetz A Gurm R Woodward AC Kline-Rogers E Leibowitz R Understanding childhood obesity in America: linkages between household income, community resources, and children’s behaviors. Am Heart J. (2012) 163(5):836–43. 10.1016/j.ahj.2012.02.025 22607862

[42] Pan L Blanck HM Sherry B Dalenius K Grummer-Strawn LM. Trends in the prevalence of extreme obesity among US preschool-aged children living in low-income families, 1998–2010. JAMA. (2012) 308(24):2563–5. 10.1001/jama.2012.108099 23268509 PMC4597777

[43] Gordon-Larsen P Adair LS Popkin BM. The relationship of ethnicity, socioeconomic factors, and overweight in US adolescents. Obes Res. (2003) 11(1):121–9. 10.1038/oby.2003.20 12529494

[44] Coleman-Jensen A Rabbitt MP Gregory CA Singh A. Household food security in the United States in 2020. Economic Research Report. 2021;No. (ERR-298) 55.

[45] Loring B Robertson A. Obesity and inequities: Guidance for addressing inequities in overweight and obesity. UN City, Marmorvej 51 DK-2100 Copenhagen Ø, Denmark: WHO Regional Office for Europe (2014).

[46] Nettle D Andrews C Bateson M. Food insecurity as a driver of obesity in humans: the insurance hypothesis. Behav Brain Sci. (2017) 40:E105. 10.1017/S0140525X16000947 PMC5266557 27464638

[47] Barriuso L Miqueleiz E Albaladejo R Villanueva R Santos JM Regidor E. Socioeconomic position and childhood-adolescent weight status in rich countries: a systematic review, 1990–2013. Bmc Pediatr. (2015) 15:129. 10.1186/s12887-015-0443-3 26391227 PMC4578240

[48] Braveman PA Cubbin C Egerter S Williams DR Pamuk E. Socioeconomic disparities in health in the United States: what the patterns tell us. Am J Public Health. (2010) 100:S186–S96. 10.2105/AJPH.2009.166082 20147693 PMC2837459

[49] Morland K Wing S Diez Roux A Poole C. Neighborhood characteristics associated with the location of food stores and food service places. Am J Prev Med. (2002) 22(1):23–9. 10.1016/S0749-3797(01)00403-2 11777675

[50] Janssen I Boyce WF Simpson K Pickett W. Influence of individual- and area-level measures of socioeconomic status on obesity, unhealthy eating, and physical inactivity in Canadian adolescents. Am J Clin Nutr. (2006) 83(1):139–45. 10.1093/ajcn/83.1.139 16400062

[51] Drewnowski A. Obesity and the food environment - dietary energy density and diet costs. Am J Prev Med. (2004) 27(3):154–62. 10.1016/j.amepre.2004.06.011 15450626

[52] Guthrie J Andrews M Frazão E Leibtag E Lin BH Mancino L Can food stamps do more to improve food choices? an economic perspective. Economic Information Bulletin. (2007) 2(EIB-29). Available at: https://www.ers.usda.gov/publications/pub-details/?pubid=44191

[53] Powell LM Slater S Chaloupka FJ Harper D. Availability of physical activity-related facilities and neighborhood demographic and socioeconomic characteristics: a national study. Am J Public Health. (2006) 96(9):1676–80. 10.2105/AJPH.2005.065573 16873753 PMC1551946

[54] Swinburn BA Sacks G Hall KD McPherson K Finegood DT Moodie ML The global obesity pandemic: shaped by global drivers and local environments. Lancet. (2011) 378(9793):804–14. 10.1016/S0140-6736(11)60813-1 21872749

[55] Scarborough P Burg MR Foster C Swinburn B Sacks G Rayner M Increased energy intake entirely accounts for increase in body weight in women but not in men in the UK between 1986 and 2000. Br J Nutr. (2011) 105(9):1399–404. 10.1017/S0007114510005076 21205425

[56] Salmon J Timperio A Telford A Carver A Crawford D. Association of family environment with children’s television viewing and with low level of physical activity. Obes Res. (2005) 13(11):1939–51. 10.1038/oby.2005.239 16339126

[57] French SA Story M Jeffery RW. Environmental influences on eating and physical activity. Annu Rev Publ Health. (2001) 22:309–35. 10.1146/annurev.publhealth.22.1.309 11274524

[58] Popkin BM Duffey K Gordon-Larsen P. Environmental influences on food choice, physical activity and energy balance. Physiol Behav. (2005) 86(5):603–13. 10.1016/j.physbeh.2005.08.051 16246381

[59] Wang Y Beydoun MA Min J Xue H Kaminsky LA Cheskin LJ. Has the prevalence of overweight, obesity and central obesity levelled off in the United States? Trends, patterns, disparities, and future projections for the obesity epidemic. Int J Epidemiol. (2020) 49(3):810–23. 10.1093/ije/dyz273 32016289 PMC7394965

[60] Barros AJ Victora CG. Measuring coverage in MNCH: determining and interpreting inequalities in coverage of maternal, newborn, and child health interventions. PLoS Med. (2013) 10(5):e1001390. 10.1371/journal.pmed.1001390 23667332 PMC3646214

[61] (WHO) WHO. Health Equity Assessment Toolkit (HEAT): Software for exploring and comparing health inequalities in countries. Built-in database edition. 2019;Version 3.1. Geneva. 10.1186/s12874-016-0229-9 PMC5069829 27760520

[62] Haider S Solon G. Life-cycle variation in the association between current and lifetime earnings. Am Econ Rev. (2006) 96(4):1308–20. 10.1257/aer.96.4.1308

[63] Hernandez DC Pressler E. Accumulation of childhood poverty on young adult overweight or obese status: race/ethnicity and gender disparities. J Epidemiol Commun H. (2014) 68(5):478–84. 10.1136/jech-2013-203062 24391207

[64] Kuczmarski RJ Ogden CL Grummer-Strawn LM Flegal KM Guo SS Wei R CDC growth charts: United States. Adv Data. (2000) 314:1–27. 11183293

[65] Group WHOMGRS. WHO child growth standards based on length/height, weight and age. Acta Paediatr Suppl. (2006) 450:76–85. 10.1111/j.1651-2227.2006.tb02378.x 16817681

